# Supplementary material for: Evaluation of a generalized knowledge-based planning performance for VMAT irradiation of breast and locoregional lymph nodes—Internal mammary and/or supraclavicular regions
Source: PLoS One. 2021 Jan 15;16(1):e0245305. doi: 10.1371/journal.pone.0245305 (PMC7810311; doi:10.1371/journal.pone.0245305)
Supplement: S1 File — (DOCX) [file pone.0245305.s001.docx]

**S1 File.**

**Identification of outliers during model’s creation**

The goodness of fit statistics refers to how well the DVH estimation model represents the training dataset. The coefficient of determination R^2^ of the regression model implies the extent of the variance explained by the regression model. Scaling from 0 to 1, a larger value indicates a better fit. The average chi square χ^2^ implies the quality of regression model, measuring the residual difference between the original and estimated data. A closer value to 1 indicates better quality of the model. Any extreme values close to 1 might represent over-fitting data [78].

The models goodness was performed by using the statistical tool embedded in RP software, where the number of possible outliers is identified in the regression of the principal components according to Cook’s distance, which is usually associated with geometric outliers and indicates influential data points in a regression model; a high value (>10) implying that the structure has a significant effect on the regression line. Whenever a single structure of a specific patient was suggested as a potential outlier, the case was carefully re-checked to look for possible odd anatomic or dosimetric features. Outliers are plans which deviate from the general trend in the analysis. Their effect on a model is typically not immediately clear because not all outliers affect the overall trend of the data in the regression analysis.

The first step in addressing identified outlying plans consisted in re-planning them. Treatment plans for which re-planning was able to reduce the dose effectively are dosimetric outliers and the outlying plans were replaced by the new plans. Treatment plans for which re-planning was unable to reduce the dose for a given OAR are geometric outliers. Careful analysis of both geometric and dosimetric outliers is an important step in RP models development. The impact of dosimetric outliers is more prominent than that of geometric outliers [60]. Nevertheless, if one outlier belongs to the geometric group, it has to be cleaned before focusing on the dosimetric ones, although it produces only a small perturbation in the regression line. Dosimetric outliers, that could be positive if they improve the model’s sparing estimates, and negative when they lie above the regression band, introduce instead large prediction variations. Negative dosimetric outliers need in general to be removed, in order to bring down the prediction uncertainty and thus improve the mean sparing estimate. Structures with a Cook’s distance >10 were also excluded.

The influence of the potential outliers was tested by removing them one by one from the training set and re-training the model. The removal of structures was done iteratively by excluding one or two strongest influential cases at the time and monitoring the improvement in the trained model. The removal phase stopped once no more significant improvement was observed. The chi-squared test was used to monitor over-fitting. A threshold value of 1.3 was used as an indication of no severe over-fitting, which means that the estimation model not only describes the training set but may generalize well for other cases. A R^2^ value too close to unity could be also a symptom of overfitting. In this case, the model would present optimal goodness-of-fit parameters but may not necessarily be able to generate good clinical treatment plans for new patients, different from the cohort used to train the model. This risk could be avoided by using a large number of initial plans on patients with a wide spectrum of anatomical differences. The features identified in a model should capture the inter-patient OAR variability, both in terms of anatomy and dose distribution. The anatomical inter-organ variability is a matter of fact, and for this reason it is fundamental to cover the largest possible knowledge on a wide anatomical spectrum. On the contrary, the dosimetric variability is a consequence of the planning strategy.

**Planning time**

Planning time was not part of the study design, since its evaluation is prone to a number of subjective or external factors not easy to quantify objectively. In particular, the experience of individual planners and workload as well as computer hardware have a strong influence. Limiting to the RP aspects, some data were collected. Once plans are identified as good candidates for the model training, the time needed to “extract” the data and load them into the configuration workspace is limited to about 15–20 s per plan. The time needed to train a model is approximately 2 min. The time needed to properly end a model configuration phase before its validation, assessing all geometric and dosimetric outlier, is very difficult to calculate and, since it is not part of the clinic, it is not here reported. Regarding the validation set and also the application of models in clinical practice, the generation of the estimated DVH and the related objectives from the model takes about 15 s and about 8–10 min for a free-run optimization, including the intermediate dose calculation phase. This shall be compared to the common time needed to prepare all the dose volume constraints, which could take several minutes. The time needed for final dose calculation is independent from the knowledge-based or conventional approach applied for optimization and depends on the algorithm and the case complexity. For breast VMAT plans it takes about 8–10 min to perform a full dose calculation with Acuros-XB.

For breast cases, the anatomy is very complicated, with many critical structures surrounding the target volume, which is usually of very irregular shape. In our current practice, many help structures were drawn to better conform the dose distribution and interactive adjustments of dose-volume constraints were always required during the optimization process. Iterative optimizations were also performed to achieve the desired dose distribution. This optimization takes 1–3 hours and depends on the complexity of the patient anatomy and the experience of the planner. The pure optimization time, in the absence of interactive intervention, is of course independent from the method chosen to generate the dose-volume constraints.

The increase in planning time if manual touch-up is needed to reach an optimum plan after RP optimization is negligible compared with the total planning time for the manual plans.

S1 Table – Overview and comparison of relevant parameters for both OARs and PTVs, averaged for the 12 patients of validation set, both for reference and model based plans all SIB. P value is reported with *, ** or *** when significant as explained in the text.

| **OAR \ Model** | **Reference SIB** | | **SIB** | | |
| --- | --- | --- | --- | --- | --- |
|  | **Average** | **Dev_st** | **Average** | **Dev_st** | **p value** |
| **Breast CNTR** | | | | | |
| **Dmax (Gy)** | 15.4 | ± 4.7 | 14.9 | ± 2.4 |  |
| **Dmean (Gy)** | 4.3 | ± 1.3 | ***4.7*** | ***± 0.9*** | ***(**)*** |
| **V_10Gy_<5%** | 3.1 | ± 2.3 | 2.8 | ± 2.0 |  |
| **Lungs** | | | | | |
| **Dmax (Gy)** | 54.8 | ± 2.2 | ***53.9*** | ***± 1.8*** | ***(***)*** |
| **Dmean (Gy)** | 9.5 | ± 1.3 | ***9.9*** | ***± 0.9*** | ***(**)*** |
| **V_5Gy_<60%** | 51.8 | ± 6.4 | ***57.1*** | ***± 6.2*** | ***(***)*** |
| **Lung IPSI** | | | | | |
| **Dmax (Gy)** | 54.8 | ± 2.2 | ***54.0*** | ***± 1.8*** | ***(***)*** |
| **Dmean (Gy)** | 14.9 | ± 2.2 | 15.6 | ± 1.2 |  |
| **V_20Gy_<40%** | 27.5 | ± 5.7 | 29.1 | ± 3.5 |  |
| **Lung CNTR** | | | | | |
| **Dmax (Gy)** | 27.5 | ± 10.3 | 27.7 | ± 9.4 |  |
| **Dmean (Gy)** | 4.1 | ± 1.1 | ***4.6*** | ***± 1.0*** | ***(**)*** |
| **V_10Gy_<5%** | 6.4 | ± 3.9 | ***8.3*** | ***± 4.4*** | ***(**)*** |
| **Heart** | | | | | |
| **Dmax (Gy)** | 34.8 | ± 15.5 | 34.1 | ± 15.4 |  |
| **Dmean (Gy)** | 5.6 | ± 2.3 | 5.8 | ± 2.3 |  |
| **V_20Gy_<10%** | 2.7 | ± 3.2 | 3.2 | ± 3.5 |  |
| **LADCA** | | | | | |
| **Dmax (Gy)** | 20.1 | ± 9.5 | 21.7 | ± 7.9 |  |
| **Dmean (Gy)** | 10.0 | ± 4.4 | ***12.3*** | ***± 4.1*** | ***(**)*** |
| **V_20Gy_<10%** | 6.8 | ± 7.3 | 7.1 | ± 6.2 |  |
| **Spinal canal** | | | | | |
| **Dmax (Gy)** | 18.0 | ± 1.6 | 19.1 | ± 1.2 |  |
| **Dmean (Gy)** | 3.7 | ± 1.0 | ***4.1*** | ***± 1.4*** | ***(**)*** |
| **Esophagus** | | | | | |
| **Dmax (Gy)** | 40.7 | ± 7.3 | 41.6 | ± 7.2 |  |
| **Thyroid** | | | | | |
| **V_40Gy_<20%** | 11.4 | ± 14.6 | 12.1 | ± 14.2 |  |
| **PTV RA** | | | | | |
| **Dmax (Gy)** | 60.2 | ± 2.5 | 59.4 | ± 1.6 |  |
| **V_95%_>95%** | 98.8 | ± 1.0 | ***98.0*** | ***± 1.0*** | ***(**)*** |
| **V_105%_<5%** | 9.9 | ± 4.8 | ***7.1*** | ***± 3.5*** | ***(***)*** |
| **PTV Surg Bed** | | | | | |
| **Dmax (Gy)** | 62.4 | ± 2.7 | 62.1 | ± 2.8 |  |
| **V_95%_>95%** | 99.5 | ± 0.5 | 99.3 | ± 0.5 |  |
| **V_105%_<5%** | 0.06 | ± 0.13 | 0.0 | ± 0.0 |  |
| **CI 100%** | | | | | |
| **V_isodose 100%_/V_PTV_** | 0.55 | ± 0.03 | ***0.57*** | ***± 0.02*** | ***(***)*** |
| **CI 95%** | | | | | |
| **V_isodose 95%_/V_PTV_** | 1.16 | ± 0.06 | ***1.11*** | ***± 0.05*** | ***(**)*** |
| **HI** | | | | | |
| **(D_2%_-D_98%_)/D_50%_** | 0.06 | ± 0.01 | 0.07 | ± 0.01 |  |
| **HI 5/95** | | | | | |
| **D_5%_/D_95%_** | 1.05 | ± 0.01 | 1.05 | ± 0.01 |  |

S1 Fig – Average DVHs for the main OARs and the PTV of the 18 patients of the internal validation set who were treated on right side. Comparison between Reference R plans in black with the square symbol and model R plans in green with the star symbol

S2 Fig – Average DVHs for the main OARs and the PTV of the 12 patients of the validation set who were treated on the left side. Comparison between Reference L plans in black with the square symbol and model L plans in green with the star symbol.

**S3 Fig – Average DVHs for the main OARs and the PTVs of the 12 patients of the validation set who were treated in SIB.** Comparison between Reference SIB plans in black with the square symbol and model B (SIB plans only) in red with the round symbol, model LN (SIB plans only) in blue with the triangle symbol, model IM LN (SIB plans only) in magenta with the rhomboidal symbol, the sum of model R and L (SIB plans only) in green with the star symbol and model SIB in cyan with the cross symbol.

**S2 Table – Percentage of outliers *a priori* and *a posteriori* of the internal validation.** *A priori* regards the outliers that the software statistic points out at the beginning of the optimization phase, red if outside the maximum value or beneath the minimum value, yellow if outwith the 90° percentile or below the 10° percentile. *A posteriori* indicates the outliers at the end of the optimization phase, which are listed for every OARs according to the QUANTEC rules, yellow if within a minor deviation, red if outside it. Each percentage for every OARs refers to the red, yellow and green values found with the software statistics or *a priori* and denotes how many outliers turn to be “real outliers” at the end of the optimization*.*

| **Model** | **B** | | | | | | | | | | | | | | | | | | | | | | | | | | | **B_No OS** | | | | | | | | | | | | | | | | | | | | | | | |
| --- | --- | --- | --- | --- | --- | --- | --- | --- | --- | --- | --- | --- | --- | --- | --- | --- | --- | --- | --- | --- | --- | --- | --- | --- | --- | --- | --- | --- | --- | --- | --- | --- | --- | --- | --- | --- | --- | --- | --- | --- | --- | --- | --- | --- | --- | --- | --- | --- | --- | --- | --- |
| **RYG a Priori** | **R** | | | | | **Y** | | | | | | **G** | | | | | | | | | | | | | | | | **R** | | | **Y** | | | | | | **G** | | | | | | | | | | | | | | |
| **RYG a Posteriori** | **G** | | | | | **G** | | | | | | **R** | | | | | **Y** | | | | | **G** | | | | | | **G** | | | **G** | | | | | | **R** | | | | | **Y** | | | | | **G** | | | | |
| **Breast CNTR** |  | | | | |  | | | | | | 17.2% | | | | | 3.4% | | | | | 79.3% | | | | | |  | | |  | | | | | | 6.9% | | | | | 6.9% | | | | | 86.2% | | | | |
| **Esophagus** |  | | | | | 100.0% | | | | | | 0.0% | | | | | 4.0% | | | | | 96.0% | | | | | |  | | | 100.0% | | | | | | 0.0% | | | | | 0.0% | | | | | 100.0% | | | | |
| **Heart** |  | | | | | 100.0% | | | | | | 0.0% | | | | | 0.0% | | | | | 100.0% | | | | | |  | | | 100.0% | | | | | | 0.0% | | | | | 0.0% | | | | | 100.0% | | | | |
| **LADCA** |  | | | | |  | | | | | | 12.9% | | | | | 6.5% | | | | | 80.6% | | | | | |  | | |  | | | | | | 9.7% | | | | | 3.2% | | | | | 87.1% | | | | |
| **Lung CNTR** |  | | | | |  | | | | | | 30.0% | | | | | 13.3% | | | | | 56.7% | | | | | |  | | |  | | | | | | 23.3% | | | | | 10.0% | | | | | 66.7% | | | | |
| **Lung IPSI** | 100.0% | | | | | 100.0% | | | | | | 0.0% | | | | | 4.1% | | | | | 95.9% | | | | | | 100.0% | | | 100.0% | | | | | | 0.0% | | | | | 3.9% | | | | | 96.1% | | | | |
| **Lungs** | 100.0% | | | | | 100.0% | | | | | | 7.1% | | | | | 0.0% | | | | | 92.9% | | | | | | 100.0% | | |  | | | | | | 3.4% | | | | | 0.0% | | | | | 96.6% | | | | |
| **Spinal canal** | 100.0% | | | | | 100.0% | | | | | | 0.0% | | | | | 4.5% | | | | | 95.5% | | | | | | 100.0% | | | 100.0% | | | | | | 0.0% | | | | | 0.0% | | | | | 100.0% | | | | |
| **Thyroid** | 100.0% | | | | | 100.0% | | | | | | 33.3% | | | | | 0.0% | | | | | 66.7% | | | | | | 100.0% | | | 100.0% | | | | | | 27.8% | | | | | 0.0% | | | | | 72.2% | | | | |
| **% of RYG a Priori** | **100.0%** | | | | | **100.0%** | | | | | | **9.2%** | | | | | **3.9%** | | | | | **87.0%** | | | | | | **100.0%** | | | **100.0%** | | | | | | **6.3%** | | | | | **2.8%** | | | | | **90.9%** | | | | |
| **Model** | **LN** | | | | | | | | | | | | | | | | | | | | | | | | | | | **LN_No OS** | | | | | | | | | | | | | | | | | | | | | | | |
| **RYG a Priori** | **R** | | | | | | | | | **Y** | | | | | **G** | | | | | | | | | | | | | **R** | | | | | **Y** | | | | | **G** | | | | | | | | | | | | | |
| **RYG a Posteriori** | **Y** | | | | **G** | | | | | **G** | | | | | **R** | | | | | **Y** | | | | | **G** | | | **G** | | | | | **G** | | | | | **R** | | | | | **Y** | | | | | **G** | | | |
| **Breast CNTR** |  | | | |  | | | | |  | | | | | 17.2% | | | | | 0.0% | | | | | 82.8% | | |  | | | | |  | | | | | 0.0% | | | | | 10.3% | | | | | 89.7% | | | |
| **Esophagus** |  | | | |  | | | | | 100.0% | | | | | 0.0% | | | | | 0.0% | | | | | 100.0% | | |  | | | | | 100.0% | | | | | 0.0% | | | | | 0.0% | | | | | 100.0% | | | |
| **Heart** |  | | | |  | | | | | 100.0% | | | | | 0.0% | | | | | 0.0% | | | | | 100.0% | | | 100.0% | | | | | 100.0% | | | | | 0.0% | | | | | 0.0% | | | | | 100.0% | | | |
| **LADCA** |  | | | |  | | | | |  | | | | | 9.7% | | | | | 6.5% | | | | | 83.9% | | |  | | | | |  | | | | | 12.9% | | | | | 0.0% | | | | | 87.1% | | | |
| **Lung CNTR** |  | | | |  | | | | |  | | | | | 40.0% | | | | | 10.0% | | | | | 50.0% | | |  | | | | |  | | | | | 30.0% | | | | | 6.7% | | | | | 63.3% | | | |
| **Lung IPSI** | 0.0% | | | | 100.0% | | | | | 100.0% | | | | | 0.0% | | | | | 4.1% | | | | | 95.9% | | | 100.0% | | | | | 100.0% | | | | | 0.0% | | | | | 3.8% | | | | | 96.2% | | | |
| **Lungs** | 0.0% | | | | 100.0% | | | | |  | | | | | 3.4% | | | | | 3.4% | | | | | 93.1% | | | 100.0% | | | | |  | | | | | 3.4% | | | | | 0.0% | | | | | 96.6% | | | |
| **Spinal canal** | 50.0% | | | | 50.0% | | | | | 100.0% | | | | | 4.5% | | | | | 9.1% | | | | | 86.4% | | | 100.0% | | | | | 100.0% | | | | | 0.0% | | | | | 4.5% | | | | | 95.5% | | | |
| **Thyroid** | 0.0% | | | | 100.0% | | | | | 100.0% | | | | | 33.3% | | | | | 0.0% | | | | | 66.7% | | | 100.0% | | | | | 100.0% | | | | | 27.8% | | | | | 0.0% | | | | | 72.2% | | | |
| **% of RYG a Priori** | **20.0%** | | | | **80.0%** | | | | | **100.0%** | | | | | **9.8%** | | | | | **3.5%** | | | | | **86.7%** | | | **100.0%** | | | | | **100.0%** | | | | | **6.6%** | | | | | **2.8%** | | | | | **90.6%** | | | |
| **Model** | **IM LN** | | | | | | | | | | | | | | | | | | | | | | | | | | | **IM LN_No OS** | | | | | | | | | | | | | | | | | | | | | | | |
| **RYG a Priori** | **R** | | | | | | | **Y** | | | | | **G** | | | | | | | | | | | | | | | **R** | | | | | | **Y** | | | | | **G** | | | | | | | | | | | | |
| **RYG a Posteriori** | **R** | | **G** | | | | | **G** | | | | | **R** | | | | | **Y** | | | | | **G** | | | | | **Y** | **G** | | | | | **G** | | | | | **R** | | | | | **Y** | | | | | **G** | | |
| **Breast CNTR** |  | |  | | | | |  | | | | | 6.9% | | | | | 10.3% | | | | | 82.8% | | | | |  |  | | | | |  | | | | | 0.0% | | | | | 3.4% | | | | | 96.6% | | |
| **Esophagus** |  | |  | | | | | 100.0% | | | | | 0.0% | | | | | 4.2% | | | | | 95.8% | | | | |  |  | | | | | 100.0% | | | | | 0.0% | | | | | 0.0% | | | | | 100.0% | | |
| **Heart** | 0.0% | | 100.0% | | | | | 100.0% | | | | | 0.0% | | | | | 0.0% | | | | | 100.0% | | | | |  |  | | | | |  | | | | | 0.0% | | | | | 0.0% | | | | | 100.0% | | |
| **LADCA** |  | |  | | | | |  | | | | | 29.0% | | | | | 0.0% | | | | | 71.0% | | | | |  |  | | | | |  | | | | | 25.8% | | | | | 0.0% | | | | | 74.2% | | |
| **Lung CNTR** |  | |  | | | | |  | | | | | 40.0% | | | | | 10.0% | | | | | 50.0% | | | | |  |  | | | | |  | | | | | 36.7% | | | | | 6.7% | | | | | 56.7% | | |
| **Lung IPSI** | 0.0% | | 100.0% | | | | | 100.0% | | | | | 0.0% | | | | | 7.8% | | | | | 92.2% | | | | | 0.0% | 100.0% | | | | | 100.0% | | | | | 0.0% | | | | | 3.8% | | | | | 96.2% | | |
| **Lungs** | 0.0% | | 100.0% | | | | |  | | | | | 14.8% | | | | | 7.4% | | | | | 77.8% | | | | | 0.0% | 100.0% | | | | |  | | | | | 3.4% | | | | | 3.4% | | | | | 93.1% | | |
| **Spinal canal** | 20.0% | | 80.0% | | | | | 100.0% | | | | | 5.3% | | | | | 15.8% | | | | | 78.9% | | | | | 16.7% | 83.3% | | | | | 100.0% | | | | | 0.0% | | | | | 10.5% | | | | | 89.5% | | |
| **Thyroid** | 0.0% | | 100.0% | | | | | 100.0% | | | | | 33.3% | | | | | 0.0% | | | | | 66.7% | | | | | 0.0% | 100.0% | | | | | 100.0% | | | | | 27.8% | | | | | 0.0% | | | | | 72.2% | | |
| **% of RYG a Priori** | **7.7%** | | **92.3%** | | | | | **100.0%** | | | | | **12.1%** | | | | | **5.7%** | | | | | **82.1%** | | | | | **11.1%** | **88.9%** | | | | | **100.0%** | | | | | **8.7%** | | | | | **2.8%** | | | | | **88.5%** | | |
| **Model** | **L** | | | | | | | | | | | | | | | | | | | | | | | | | | **L_No OS** | | | | | | | | | | | | | | | | | | | | | | | | |
| **RYG a Priori** | **R** | | | | | | | | **Y** | | | | | **G** | | | | | | | | | | | | | **R** | | | | | | | | **Y** | | | | | **G** | | | | | | | | | | | |
| **RYG a Posteriori** | **R** | | | **G** | | | | | **G** | | | | | **R** | | | | | **Y** | | | | | **G** | | | **Y** | | | **G** | | | | | **G** | | | | | **R** | | | | | **Y** | | | | | **G** | |
| **Breast CNTR** |  | | |  | | | | |  | | | | | 9.1% | | | | | 18.2% | | | | | 72.7% | | |  | | |  | | | | |  | | | | | 9.1% | | | | | 0.0% | | | | | 90.9% | |
| **Esophagus** |  | | |  | | | | | 100.0% | | | | | 0.0% | | | | | 10.0% | | | | | 90.0% | | |  | | |  | | | | | 100.0% | | | | | 0.0% | | | | | 0.0% | | | | | 100.0% | |
| **Heart** |  | | |  | | | | |  | | | | | 0.0% | | | | | 0.0% | | | | | 100.0% | | |  | | |  | | | | | 100.0% | | | | | 0.0% | | | | | 0.0% | | | | | 100.0% | |
| **LADCA** |  | | |  | | | | |  | | | | | 22.2% | | | | | 0.0% | | | | | 77.8% | | |  | | |  | | | | |  | | | | | 18.5% | | | | | 3.7% | | | | | 77.8% | |
| **Lung CNTR** | 100.0% | | | 0.0% | | | | |  | | | | | 90.9% | | | | | 0.0% | | | | | 9.1% | | | 100.0% | | | 0.0% | | | | |  | | | | | 72.7% | | | | | 18.2% | | | | | 9.1% | |
| **Lung IPSI** |  | | |  | | | | | 100.0% | | | | | 0.0% | | | | | 0.0% | | | | | 100.0% | | |  | | |  | | | | | 100.0% | | | | | 0.0% | | | | | 0.0% | | | | | 100.0% | |
| **Lungs** |  | | |  | | | | |  | | | | | 8.3% | | | | | 0.0% | | | | | 91.7% | | |  | | |  | | | | |  | | | | | 0.0% | | | | | 0.0% | | | | | 100.0% | |
| **Spinal canal** | 0.0% | | | 100.0% | | | | |  | | | | | 0.0% | | | | | 22.2% | | | | | 77.8% | | | 0.0% | | | 100.0% | | | | |  | | | | | 0.0% | | | | | 0.0% | | | | | 100.0% | |
| **Thyroid** |  | | |  | | | | | 100.0% | | | | | 37.5% | | | | | 0.0% | | | | | 62.5% | | |  | | |  | | | | | 100.0% | | | | | 37.5% | | | | | 0.0% | | | | | 62.5% | |
| **% of RYG a Priori** | **33.3%** | | | **66.7%** | | | | | **100.0%** | | | | | **15.6%** | | | | | **3.7%** | | | | | **80.7%** | | | **25.0%** | | | **75.0%** | | | | | **100.0%** | | | | | **12.8%** | | | | | **2.3%** | | | | | **85.0%** | |
| **Model** | **R** | | | | | | | | | | | | | | | | | | | | | | | | | | **R_No OS** | | | | | | | | | | | | | | | | | | | | | | | | |
| **RYG a Priori** | **R** | | | | | | | | | | **Y** | | | | | **G** | | | | | | | | | | | **R** | | | | | **Y** | | | | | | | | | **G** | | | | | | | | | | |
| **RYG a Posteriori** | **R** | **Y** | | | | | **G** | | | | **G** | | | | | **R** | | | | | **Y** | | | | | **G** | **G** | | | | | **Y** | | | | **G** | | | | | **R** | | | | | **Y** | | | | | **G** |
| **Breast CNTR** |  |  | | | | |  | | | |  | | | | | 22.2% | | | | | 0.0% | | | | | 77.8% |  | | | | |  | | | |  | | | | | 0.0% | | | | | 5.6% | | | | | 94.4% |
| **Esophagus** |  |  | | | | |  | | | | 100.0% | | | | | 0.0% | | | | | 0.0% | | | | | 100.0% |  | | | | | 0.0% | | | | 100.0% | | | | | 0.0% | | | | | 0.0% | | | | | 100.0% |
| **Heart** | 0.0% | 0.0% | | | | | 100.0% | | | | 100.0% | | | | | 0.0% | | | | | 0.0% | | | | | 100.0% | 100.0% | | | | |  | | | |  | | | | | 0.0% | | | | | 0.0% | | | | | 100.0% |
| **LADCA** |  |  | | | | |  | | | |  | | | | | 0.0% | | | | | 0.0% | | | | | 100.0% |  | | | | |  | | | |  | | | | | 0.0% | | | | | 0.0% | | | | | 100.0% |
| **Lung CNTR** |  |  | | | | |  | | | |  | | | | | 16.7% | | | | | 5.6% | | | | | 77.8% |  | | | | |  | | | |  | | | | | 5.6% | | | | | 5.6% | | | | | 88.9% |
| **Lung IPSI** | 0.0% | 0.0% | | | | | 100.0% | | | | 100.0% | | | | | 6.1% | | | | | 6.1% | | | | | 87.9% | 100.0% | | | | | 0.0% | | | | 100.0% | | | | | 6.7% | | | | | 3.3% | | | | | 90.0% |
| **Lungs** | 100.0% | 0.0% | | | | | 0.0% | | | | 100.0% | | | | | 21.4% | | | | | 7.1% | | | | | 71.4% | 100.0% | | | | | 33.3% | | | | 66.7% | | | | | 14.3% | | | | | 7.1% | | | | | 78.6% |
| **Spinal canal** | 0.0% | 50.0% | | | | | 50.0% | | | | 100.0% | | | | | 8.3% | | | | | 0.0% | | | | | 91.7% | 100.0% | | | | | 0.0% | | | | 100.0% | | | | | 0.0% | | | | | 0.0% | | | | | 100.0% |
| **Thyroid** | 0.0% | 0.0% | | | | | 100.0% | | | |  | | | | | 20.0% | | | | | 10.0% | | | | | 70.0% | 100.0% | | | | |  | | | |  | | | | | 30.0% | | | | | 0.0% | | | | | 70.0% |
| **% of RYG a Priori** | **16.7%** | **16.7%** | | | | | **66.7%** | | | | **100.0%** | | | | | **10.1%** | | | | | **3.4%** | | | | | **86.6%** | **100.0%** | | | | | **10.0%** | | | | **90.0%** | | | | | **5.4%** | | | | | **2.7%** | | | | | **91.9%** |

**S3 Table – Percentage of outliers after the internal validation with respect to the reference plans.** *RYG Ref* regards the constraints in the Reference plans that, according to the QUANTEC rules, are yellow if in minor deviation, red if outside it. *RYG Model* refers to the parameters after the optimization model-based phase, listed for every OARs, that turn to be green, yellow, or red. Each percentage refers to the red, yellow and green values denotes how many outliers changed respect to the reference plans*.*

| **Model** | | | | | **B** | | | | | | | | | | | | | | | | | | | | | | | | | | | | | | | | | | | | | | **B_No OS** | | | | | | | | | | | | | | | | | | | | | | | | | | | | | | | | |
| --- | --- | --- | --- | --- | --- | --- | --- | --- | --- | --- | --- | --- | --- | --- | --- | --- | --- | --- | --- | --- | --- | --- | --- | --- | --- | --- | --- | --- | --- | --- | --- | --- | --- | --- | --- | --- | --- | --- | --- | --- | --- | --- | --- | --- | --- | --- | --- | --- | --- | --- | --- | --- | --- | --- | --- | --- | --- | --- | --- | --- | --- | --- | --- | --- | --- | --- | --- | --- | --- | --- | --- | --- | --- | --- | --- |
| **RYG Ref** | | | | | **R** | | | | | | | | | | | | | **Y** | | | | | | | | | | | | | | | | **G** | | | | | | | | | **R** | | | | | | | | | | | | | | **Y** | | | | | | | | | | **G** | | | | | | | | |
| **RYG Model** | | | | | **R** | | **Y** | | | | | **G** | | | | | | **R** | | | | | **Y** | | | | | | **G** | | | | | **R** | | | **Y** | | | **G** | | | **R** | | | | | **Y** | | | | | | **G** | | | **Y** | | | | **G** | | | | | | **R** | | | | | **Y** | | | **G** |
| **Breast CNTR** | | | | | 100.0% | | 0.0% | | | | | 0.0% | | | | | | 0.0% | | | | | 0.0% | | | | | | 100.0% | | | | | 14.8% | | | 3.7% | | | 81.5% | | | 100.0% | | | | | 0.0% | | | | | | 0.0% | | | 100.0% | | | | 0.0% | | | | | | 3.7% | | | | | 3.7% | | | 92.6% |
| **Esophagus** | | | | | 0.0% | | 100.0% | | | | | 0.0% | | | | | |  | | | | |  | | | | | |  | | | | | 0.0% | | | 0.0% | | | 100.0% | | | 0.0% | | | | | 0.0% | | | | | | 100.0% | | |  | | | |  | | | | | | 0.0% | | | | | 0.0% | | | 100.0% |
| **Heart** | | | | | 0.0% | | 0.0% | | | | | 100.0% | | | | | |  | | | | |  | | | | | |  | | | | | 0.0% | | | 0.0% | | | 100.0% | | | 0.0% | | | | | 0.0% | | | | | | 100.0% | | |  | | | |  | | | | | | 0.0% | | | | | 0.0% | | | 100.0% |
| **LADCA** | | | | | 100.0% | | 0.0% | | | | | 0.0% | | | | | | 0.0% | | | | | 100.0% | | | | | | 0.0% | | | | | 0.0% | | | 3.8% | | | 96.2% | | | 75.0% | | | | | 0.0% | | | | | | 25.0% | | | 100.0% | | | | 0.0% | | | | | | 0.0% | | | | | 0.0% | | | 100.0% |
| **Lung CNTR** | | | | | 70.0% | | 10.0% | | | | | 20.0% | | | | | | 100.0% | | | | | 0.0% | | | | | | 0.0% | | | | | 5.3% | | | 15.8% | | | 78.9% | | | 60.0% | | | | | 10.0% | | | | | | 30.0% | | | 100.0% | | | | 0.0% | | | | | | 5.3% | | | | | 5.3% | | | 89.5% |
| **Lung IPSI** | | | | | 0.0% | | 0.0% | | | | | 100.0% | | | | | | 0.0% | | | | | 0.0% | | | | | | 100.0% | | | | | 0.0% | | | 3.6% | | | 96.4% | | | 0.0% | | | | | 0.0% | | | | | | 100.0% | | | 0.0% | | | | 100.0% | | | | | | 0.0% | | | | | 3.6% | | | 96.4% |
| **Lungs** | | | | | 50.0% | | 0.0% | | | | | 50.0% | | | | | | 0.0% | | | | | 0.0% | | | | | | 100.0% | | | | | 3.7% | | | 0.0% | | | 96.3% | | | 50.0% | | | | | 0.0% | | | | | | 50.0% | | | 0.0% | | | | 100.0% | | | | | | 0.0% | | | | | 0.0% | | | 100.0% |
| **Spinal canal** | | | | | 0.0% | | 0.0% | | | | | 100.0% | | | | | |  | | | | |  | | | | | |  | | | | | 0.0% | | | 4.2% | | | 95.8% | | | 0.0% | | | | | 0.0% | | | | | | 100.0% | | |  | | | |  | | | | | | 0.0% | | | | | 0.0% | | | 100.0% |
| **Thyroid** | | | | | 83.3% | | 0.0% | | | | | 16.7% | | | | | |  | | | | |  | | | | | |  | | | | | 7.1% | | | 0.0% | | | 92.9% | | | 83.3% | | | | | 0.0% | | | | | | 16.7% | | |  | | | |  | | | | | | 0.0% | | | | | 0.0% | | | 100.0% |
| **Tot** | | | | | **62.1%** | | **6.9%** | | | | | **31.0%** | | | | | | **16.7%** | | | | | **16.7%** | | | | | | **66.7%** | | | | | **2.6%** | | | **3.0%** | | | **94.4%** | | | **55.2%** | | | | | **3.4%** | | | | | | **41.4%** | | | **50.0%** | | | | **50.0%** | | | | | | **0.7%** | | | | | **1.5%** | | | **97.8%** |
| **Model** | | | | | **LN** | | | | | | | | | | | | | | | | | | | | | | | | | | | | | | | | | | | **LN_No OS** | | | | | | | | | | | | | | | | | | | | | | | | | | | | | | | | | | | |
| **RYG Ref** | | | | | **R** | | | | | | | | | | | **Y** | | | | | | | | | **G** | | | | | | | | | | | | | | | **R** | | | | | | | | | | | | | **Y** | | | | | | | | | | | | | **G** | | | | | | | | | |
| **RYG Model** | | | | | **R** | | **Y** | | | **G** | | | | | | **R** | | | | | **G** | | | | **R** | | | | | **Y** | | | | | **G** | | | | | **R** | | **Y** | | | | **G** | | | | | | | **R** | | | **Y** | | | | **G** | | | | | | **R** | | | | **Y** | | | | | **G** |
| **Breast CNTR** | | | | | 0.0% | | 0.0% | | | 100.0% | | | | | | 100.0% | | | | | 0.0% | | | | 14.8% | | | | | 0.0% | | | | | 85.2% | | | | | 0.0% | | 100.0% | | | | 0.0% | | | | | | | 0.0% | | | 100.0% | | | | 0.0% | | | | | | 0.0% | | | | 3.7% | | | | | 96.3% |
| **Esophagus** | | | | | 0.0% | | 0.0% | | | 100.0% | | | | | |  | | | | |  | | | | 0.0% | | | | | 0.0% | | | | | 100.0% | | | | | 0.0% | | 0.0% | | | | 100.0% | | | | | | |  | | |  | | | |  | | | | | | 0.0% | | | | 0.0% | | | | | 100.0% |
| **Heart** | | | | | 0.0% | | 0.0% | | | 100.0% | | | | | |  | | | | |  | | | | 0.0% | | | | | 0.0% | | | | | 100.0% | | | | | 0.0% | | 0.0% | | | | 100.0% | | | | | | |  | | |  | | | |  | | | | | | 0.0% | | | | 0.0% | | | | | 100.0% |
| **LADCA** | | | | | 75.0% | | 25.0% | | | 0.0% | | | | | | 0.0% | | | | | 100.0% | | | | 0.0% | | | | | 3.8% | | | | | 96.2% | | | | | 75.0% | | 0.0% | | | | 25.0% | | | | | | | 0.0% | | | 0.0% | | | | 100.0% | | | | | | 3.8% | | | | 0.0% | | | | | 96.2% |
| **Lung CNTR** | | | | | 80.0% | | 0.0% | | | 20.0% | | | | | | 100.0% | | | | | 0.0% | | | | 15.8% | | | | | 15.8% | | | | | 68.4% | | | | | 70.0% | | 0.0% | | | | 30.0% | | | | | | | 100.0% | | | 0.0% | | | | 0.0% | | | | | | 5.3% | | | | 10.5% | | | | | 84.2% |
| **Lung IPSI** | | | | | 0.0% | | 0.0% | | | 100.0% | | | | | | 0.0% | | | | | 100.0% | | | | 0.0% | | | | | 3.6% | | | | | 96.4% | | | | | 0.0% | | 0.0% | | | | 100.0% | | | | | | | 0.0% | | | 0.0% | | | | 100.0% | | | | | | 0.0% | | | | 3.6% | | | | | 96.4% |
| **Lungs** | | | | | 50.0% | | 0.0% | | | 50.0% | | | | | | 0.0% | | | | | 100.0% | | | | 0.0% | | | | | 3.7% | | | | | 96.3% | | | | | 50.0% | | 0.0% | | | | 50.0% | | | | | | | 0.0% | | | 0.0% | | | | 100.0% | | | | | | 0.0% | | | | 0.0% | | | | | 100.0% |
| **Spinal canal** | | | | | 50.0% | | 0.0% | | | 50.0% | | | | | |  | | | | |  | | | | 0.0% | | | | | 12.5% | | | | | 87.5% | | | | | 0.0% | | 50.0% | | | | 50.0% | | | | | | |  | | |  | | | |  | | | | | | 0.0% | | | | 0.0% | | | | | 100.0% |
| **Thyroid** | | | | | 83.3% | | 0.0% | | | 16.7% | | | | | |  | | | | |  | | | | 7.1% | | | | | 0.0% | | | | | 92.9% | | | | | 83.3% | | 0.0% | | | | 16.7% | | | | | | |  | | |  | | | |  | | | | | | 0.0% | | | | 0.0% | | | | | 100.0% |
| **Tot** | | | | | **62.1%** | | **3.4%** | | | **34.5%** | | | | | | **33.3%** | | | | | **66.7%** | | | | **3.0%** | | | | | **3.7%** | | | | | **93.3%** | | | | | **55.2%** | | **6.9%** | | | | **37.9%** | | | | | | | **16.7%** | | | **16.7%** | | | | **66.7%** | | | | | | **0.7%** | | | | **1.9%** | | | | | **97.4%** |
| **Model** | | | | **IM LN** | | | | | | | | | | | | | | | | | | | | | | | | | | | | | | | | | | | | | | **IM LN_No OS** | | | | | | | | | | | | | | | | | | | | | | | | | | | | | | | | | |
| **RYG Ref** | | | | **R** | | | | | | | | | | | | | **Y** | | | | | | | | | | | | | | | | **G** | | | | | | | | | **R** | | | | | | | | | | | | | **Y** | | | | | | | | | | **G** | | | | | | | | | | |
| **RYG Model** | | | | **R** | | **Y** | | | | | **G** | | | | | | **R** | | | | | **Y** | | | | | | **G** | | | | | **R** | | | **Y** | | | | **G** | | **R** | | | | | **Y** | | | | **G** | | | | **R** | | | | **G** | | | | | | **R** | | | | | | **Y** | | | | **G** |
| **Breast CNTR** | | | | 100.0% | | 0.0% | | | | | 0.0% | | | | | | 0.0% | | | | | 100.0% | | | | | | 0.0% | | | | | 3.7% | | | 7.4% | | | | 88.9% | | 0.0% | | | | | 0.0% | | | | 100.0% | | | | 0.0% | | | | 100.0% | | | | | | 0.0% | | | | | | 3.7% | | | | 96.3% |
| **Esophagus** | | | | 0.0% | | 100.0% | | | | | 0.0% | | | | | |  | | | | |  | | | | | |  | | | | | 0.0% | | | 0.0% | | | | 100.0% | | 0.0% | | | | | 0.0% | | | | 100.0% | | | |  | | | |  | | | | | | 0.0% | | | | | | 0.0% | | | | 100.0% |
| **Heart** | | | | 0.0% | | 0.0% | | | | | 100.0% | | | | | |  | | | | |  | | | | | |  | | | | | 0.0% | | | 0.0% | | | | 100.0% | | 0.0% | | | | | 0.0% | | | | 100.0% | | | |  | | | |  | | | | | | 0.0% | | | | | | 0.0% | | | | 100.0% |
| **LADCA** | | | | 100.0% | | 0.0% | | | | | 0.0% | | | | | | 0.0% | | | | | 0.0% | | | | | | 100.0% | | | | | 19.2% | | | 0.0% | | | | 80.8% | | 100.0% | | | | | 0.0% | | | | 0.0% | | | | 0.0% | | | | 100.0% | | | | | | 15.4% | | | | | | 0.0% | | | | 84.6% |
| **Lung CNTR** | | | | 70.0% | | 10.0% | | | | | 20.0% | | | | | | 100.0% | | | | | 0.0% | | | | | | 0.0% | | | | | 21.1% | | | 10.5% | | | | 68.4% | | 80.0% | | | | | 0.0% | | | | 20.0% | | | | 100.0% | | | | 0.0% | | | | | | 10.5% | | | | | | 10.5% | | | | 78.9% |
| **Lung IPSI** | | | | 0.0% | | 0.0% | | | | | 100.0% | | | | | | 0.0% | | | | | 0.0% | | | | | | 100.0% | | | | | 0.0% | | | 7.1% | | | | 92.9% | | 0.0% | | | | | 0.0% | | | | 100.0% | | | | 0.0% | | | | 100.0% | | | | | | 0.0% | | | | | | 3.6% | | | | 96.4% |
| **Lungs** | | | | 50.0% | | 50.0% | | | | | 0.0% | | | | | | 0.0% | | | | | 0.0% | | | | | | 100.0% | | | | | 11.1% | | | 3.7% | | | | 85.2% | | 50.0% | | | | | 0.0% | | | | 50.0% | | | | 0.0% | | | | 100.0% | | | | | | 0.0% | | | | | | 3.7% | | | | 96.3% |
| **Spinal canal** | | | | 0.0% | | 50.0% | | | | | 50.0% | | | | | |  | | | | |  | | | | | |  | | | | | 8.3% | | | 8.3% | | | | 83.3% | | 0.0% | | | | | 50.0% | | | | 50.0% | | | |  | | | |  | | | | | | 0.0% | | | | | | 8.3% | | | | 91.7% |
| **Thyroid** | | | | 83.3% | | 0.0% | | | | | 16.7% | | | | | |  | | | | |  | | | | | |  | | | | | 7.1% | | | 0.0% | | | | 92.9% | | 83.3% | | | | | 0.0% | | | | 16.7% | | | |  | | | |  | | | | | | 0.0% | | | | | | 0.0% | | | | 100.0% |
| **Tot** | | | | **62.1%** | | **13.8%** | | | | | **24.1%** | | | | | | **16.7%** | | | | | **16.7%** | | | | | | **66.7%** | | | | | **5.9%** | | | **4.1%** | | | | **90.0%** | | **62.1%** | | | | | **3.4%** | | | | **34.5%** | | | | **16.7%** | | | | **83.3%** | | | | | | **2.2%** | | | | | | **3.0%** | | | | **94.8%** |
| **Model** | | | **L** | | | | | | | | | | | | | | | | | | | | | | | | | | | | | | | | | | | | | | | **L_No OS** | | | | | | | | | | | | | | | | | | | | | | | | | | | | | | | | | |
| **RYG Ref** | | | **R** | | | | | | | | | | | | | | | | **Y** | | | | | | | **G** | | | | | | | | | | | | | | | | **R** | | | | | | | | | | | | | | | **Y** | | | | | **G** | | | | | | | | | | | | | |
| **RYG Model** | | | **R** | | | | | | **Y** | | | | | **G** | | | | | **R** | | | | | | | **R** | | | | | **Y** | | | | | | | **G** | | | | **R** | | | **Y** | | | | **G** | | | | | | | | **R** | | | | | **R** | | | | | | | **Y** | | | | | **G** | |
| **Breast CNTR** | | | 100.0% | | | | | | 0.0% | | | | | 0.0% | | | | |  | | | | | | | 0.0% | | | | | 20.0% | | | | | | | 80.0% | | | | 100.0% | | | 0.0% | | | | 0.0% | | | | | | | |  | | | | | 0.0% | | | | | | | 0.0% | | | | | 100.0% | |
| **Esophagus** | | | 0.0% | | | | | | 100.0% | | | | | 0.0% | | | | |  | | | | | | | 0.0% | | | | | 0.0% | | | | | | | 100.0% | | | | 0.0% | | | 0.0% | | | | 100.0% | | | | | | | |  | | | | | 0.0% | | | | | | | 0.0% | | | | | 100.0% | |
| **Heart** | | | 0.0% | | | | | | 0.0% | | | | | 100.0% | | | | |  | | | | | | | 0.0% | | | | | 0.0% | | | | | | | 100.0% | | | | 0.0% | | | 0.0% | | | | 100.0% | | | | | | | |  | | | | | 0.0% | | | | | | | 0.0% | | | | | 100.0% | |
| **LADCA** | | | 100.0% | | | | | | 0.0% | | | | | 0.0% | | | | | 100.0% | | | | | | | 4.5% | | | | | 0.0% | | | | | | | 95.5% | | | | 75.0% | | | 25.0% | | | | 0.0% | | | | | | | | 100.0% | | | | | 4.5% | | | | | | | 0.0% | | | | | 95.5% | |
| **Lung CNTR** | | | 100.0% | | | | | | 0.0% | | | | | 0.0% | | | | | 100.0% | | | | | | | 66.7% | | | | | 0.0% | | | | | | | 33.3% | | | | 87.5% | | | 12.5% | | | | 0.0% | | | | | | | | 100.0% | | | | | 33.3% | | | | | | | 33.3% | | | | | 33.3% | |
| **Lung IPSI** | | | 0.0% | | | | | | 0.0% | | | | | 100.0% | | | | |  | | | | | | | 0.0% | | | | | 0.0% | | | | | | | 100.0% | | | | 0.0% | | | 0.0% | | | | 100.0% | | | | | | | |  | | | | | 0.0% | | | | | | | 0.0% | | | | | 100.0% | |
| **Lungs** | | | 0.0% | | | | | | 0.0% | | | | | 100.0% | | | | |  | | | | | | | 9.1% | | | | | 0.0% | | | | | | | 90.9% | | | | 0.0% | | | 0.0% | | | | 100.0% | | | | | | | |  | | | | | 0.0% | | | | | | | 0.0% | | | | | 100.0% | |
| **Spinal canal** | | | 0.0% | | | | | | 0.0% | | | | | 100.0% | | | | |  | | | | | | | 0.0% | | | | | 20.0% | | | | | | | 80.0% | | | | 0.0% | | | 0.0% | | | | 100.0% | | | | | | | |  | | | | | 0.0% | | | | | | | 0.0% | | | | | 100.0% | |
| **Thyroid** | | | 100.0% | | | | | | 0.0% | | | | | 0.0% | | | | |  | | | | | | | 0.0% | | | | | 0.0% | | | | | | | 100.0% | | | | 100.0% | | | 0.0% | | | | 0.0% | | | | | | | |  | | | | | 0.0% | | | | | | | 0.0% | | | | | 100.0% | |
| **Tot** | | | **72.7%** | | | | | | **4.5%** | | | | | **22.7%** | | | | | **100.0%** | | | | | | | **3.4%** | | | | | **3.4%** | | | | | | | **93.2%** | | | | **63.6%** | | | **9.1%** | | | | **27.3%** | | | | | | | | **100.0%** | | | | | **1.7%** | | | | | | | **0.9%** | | | | | **97.4%** | |
| **Model** | | | **R** | | | | | | | | | | | | | | | | | | | | | | | | | | | | | | | | | | | | | | | **R_No OS** | | | | | | | | | | | | | | | | | | | | | | | | | | | | | | | | | |
| **RYG Ref** | **R** | | | | | | | | | | | | **Y** | | | | | | | | | | | **G** | | | | | | | | | | | | | | | | | | **R** | | | | | | | | | | **Y** | | | | | | | | | | | | **G** | | | | | | | | | | | |
| **RYG Model** | | **R** | | | | | | **G** | | | | | | | **R** | | | | | **G** | | | | | | | **R** | | | | | **Y** | | | | | | | **G** | | **R** | | | **G** | | | | | | **R** | | | | | | | | **G** | | | | | **R** | | | | | **Y** | | | | | **G** | | |
| **Breast CNTR** | |  | | | | | |  | | | | | | | 100.0% | | | | | 0.0% | | | | | | | 17.6% | | | | | 0.0% | | | | | | | 82.4% | |  | | |  | | | | | | 0.0% | | | | | | | | 100.0% | | | | | 0.0% | | | | | 5.9% | | | | | 94.1% | | |
| **Esophagus** | |  | | | | | |  | | | | | | |  | | | | |  | | | | | | | 0.0% | | | | | 0.0% | | | | | | | 100.0% | |  | | |  | | | | | |  | | | | | | | |  | | | | | 0.0% | | | | | 0.0% | | | | | 100.0% | | |
| **Heart** | |  | | | | | |  | | | | | | |  | | | | |  | | | | | | | 0.0% | | | | | 0.0% | | | | | | | 100.0% | |  | | |  | | | | | |  | | | | | | | |  | | | | | 0.0% | | | | | 0.0% | | | | | 100.0% | | |
| **LADCA** | |  | | | | | |  | | | | | | |  | | | | |  | | | | | | | 0.0% | | | | | 0.0% | | | | | | | 100.0% | |  | | |  | | | | | |  | | | | | | | |  | | | | | 0.0% | | | | | 0.0% | | | | | 100.0% | | |
| **Lung CNTR** | | 0.0% | | | | | | 100.0% | | | | | | |  | | | | |  | | | | | | | 18.8% | | | | | 6.3% | | | | | | | 75.0% | | 0.0% | | | 100.0% | | | | | |  | | | | | | | |  | | | | | 6.3% | | | | | 6.3% | | | | | 87.5% | | |
| **Lung IPSI** | |  | | | | | |  | | | | | | | 0.0% | | | | | 100.0% | | | | | | | 5.9% | | | | | 5.9% | | | | | | | 88.2% | |  | | |  | | | | | | 0.0% | | | | | | | | 100.0% | | | | | 5.9% | | | | | 2.9% | | | | | 91.2% | | |
| **Lungs** | | 100.0% | | | | | | 0.0% | | | | | | | 100.0% | | | | | 0.0% | | | | | | | 12.5% | | | | | 6.3% | | | | | | | 81.3% | | 100.0% | | | 0.0% | | | | | | 100.0% | | | | | | | | 0.0% | | | | | 0.0% | | | | | 12.5% | | | | | 87.5% | | |
| **Spinal canal** | | 100.0% | | | | | | 0.0% | | | | | | |  | | | | |  | | | | | | | 0.0% | | | | | 7.1% | | | | | | | 92.9% | | 0.0% | | | 100.0% | | | | | |  | | | | | | | |  | | | | | 0.0% | | | | | 0.0% | | | | | 100.0% | | |
| **Thyroid** | | 66.7% | | | | | | 33.3% | | | | | | |  | | | | |  | | | | | | | 0.0% | | | | | 12.5% | | | | | | | 87.5% | | 66.7% | | | 33.3% | | | | | |  | | | | | | | |  | | | | | 12.5% | | | | | 0.0% | | | | | 87.5% | | |
| **Tot** | | **57.1%** | | | | | | **42.9%** | | | | | | | **50.0%** | | | | | **50.0%** | | | | | | | **6.5%** | | | | | **3.9%** | | | | | | | **89.5%** | | **42.9%** | | | **57.1%** | | | | | | **25.0%** | | | | | | | | **75.0%** | | | | | **2.6%** | | | | | **3.3%** | | | | | **94.1%** | | |

S4 Table – Percentage of outliers that are highlighted at the beginning of the estimation of the DVHs for each of the 10 patients of the external validation model set for each OAR for each model that has been validated from the clinic 2. Red outliers are so flagged if they are over the maximum value or under the minimum value; while yellow outliers are outwith the 90° percentile or beneath the 10° percentile but still under the maximum value or over the minimum value of the box plots.

| **Model** | **B_No OS** | **R_No OS** |
| --- | --- | --- |
| **Tot** | | |
| **R** | 39.8% | 25.2% |
| **Y** | 4.0% | 5.5% |
| **G** | 56.2% | 69.3% |
| **Breast L** | | |
| **R** | 44.3% | 32.9% |
| **Y** | 8.6% | 1.4% |
| **G** | 47.1% | 65.7% |
| **Heart** | | |
| **R** | 40.0% | 22.9% |
| **Y** | 1.4% | 1.4% |
| **G** | 58.6% | 75.7% |
| **Lung CNTR** | | |
| **R** | 40.0% | 31.4% |
| **Y** | 1.4% | 0.0% |
| **G** | 58.6% | 68.6% |
| **Lung IPSI** | | |
| **R** | 27.1% | 21.4% |
| **Y** | 0.0% | 17.1% |
| **G** | 72.9% | 61.4% |
| **Lungs** | | |
| **R** | 48.6% | 21.4% |
| **Y** | 10.0% | 8.6% |
| **G** | 41.4% | 70.0% |
| **Spinal Canal** | | |
| **R** | 38.6% | 21.4% |
| **Y** | 2.9% | 4.3% |
| **G** | 58.6% | 74.3% |

S5 Table – Percentage of outliers for each parameter, either geometrical or statistical, that are taken into account at the beginning of the estimation of the DVHs for each of the 10 patients of the external validation model set for each model that has been validated from the clinic 2. Red outliers are so flagged if they are over the maximum value or under the minimum value; while yellow outliers are outwith the 90° percentile or beneath the 10° percentile but still under the maximum value or over the minimum value of the box plots.

| **Model** | **B_No OS** | **R_No OS** |
| --- | --- | --- |
| **Tot** | | |
| **R** | 39.8% | 25.2% |
| **Y** | 4.0% | 5.5% |
| **G** | 56.2% | 69.3% |
| **Volume** | | |
| **R** | 8.3% | 11.7% |
| **Y** | 1.7% | 3.3% |
| **G** | 90.0% | 85.0% |
| **Joint target volume** | | |
| **R** | 88.3% | 33.3% |
| **Y** | 0.0% | 0.0% |
| **G** | 11.7% | 66.7% |
| **Out of field volume** | | |
| **R** | 86.7% | 60.0% |
| **Y** | 3.3% | 0.0% |
| **G** | 10.0% | 40.0% |
| **Overlap volume with targets** | | |
| **R** | 0.0% | 0.0% |
| **Y** | 0.0% | 10.0% |
| **G** | 100.0% | 90.0% |
| **PCS1** | | |
| **R** | 78.3% | 61.7% |
| **Y** | 1.7% | 18.3% |
| **G** | 20.0% | 20.0% |
| **PCS2** | | |
| **R** | 16.7% | 10.0% |
| **Y** | 13.3% | 6.7% |
| **G** | 70.0% | 83.3% |
| **PCS3** | | |
| **R** | 0.0% | 0.0% |
| **Y** | 8.3% | 0.0% |
| **G** | 91.7% | 100.0% |
