# Supplementary figures and images for: Evaluation of a generalized knowledge-based planning performance for VMAT irradiation of breast and locoregional lymph nodes—Internal mammary and/or supraclavicular regions
Source: PLoS One. 2021 Jan 15;16(1):e0245305. doi: 10.1371/journal.pone.0245305 (PMC7810311; doi:10.1371/journal.pone.0245305)

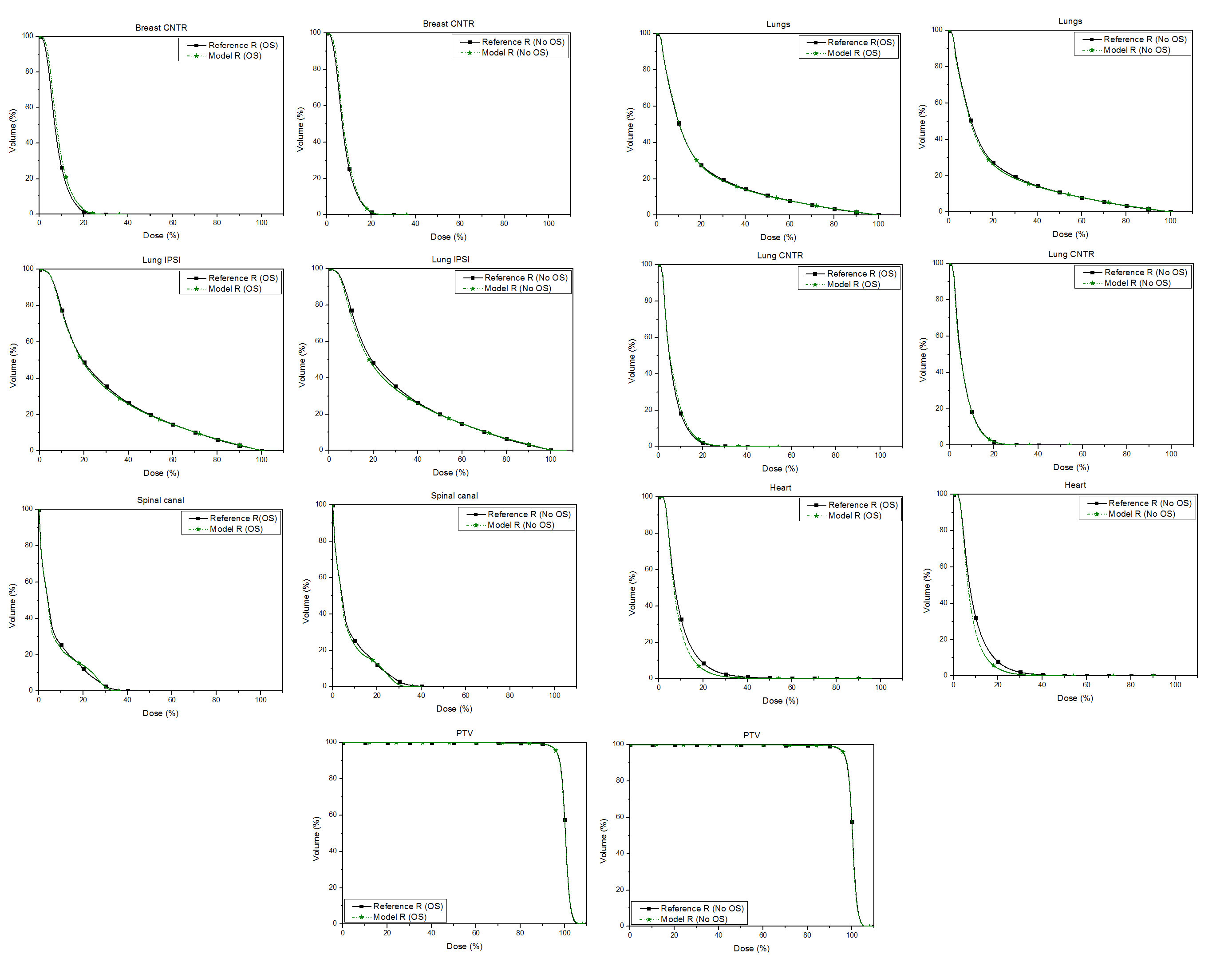

Supplement: S1 Fig — Comparison between Reference R plans in black with the square symbol and model R plans in green with the star symbol. (TIF) [file pone.0245305.s002.tif]

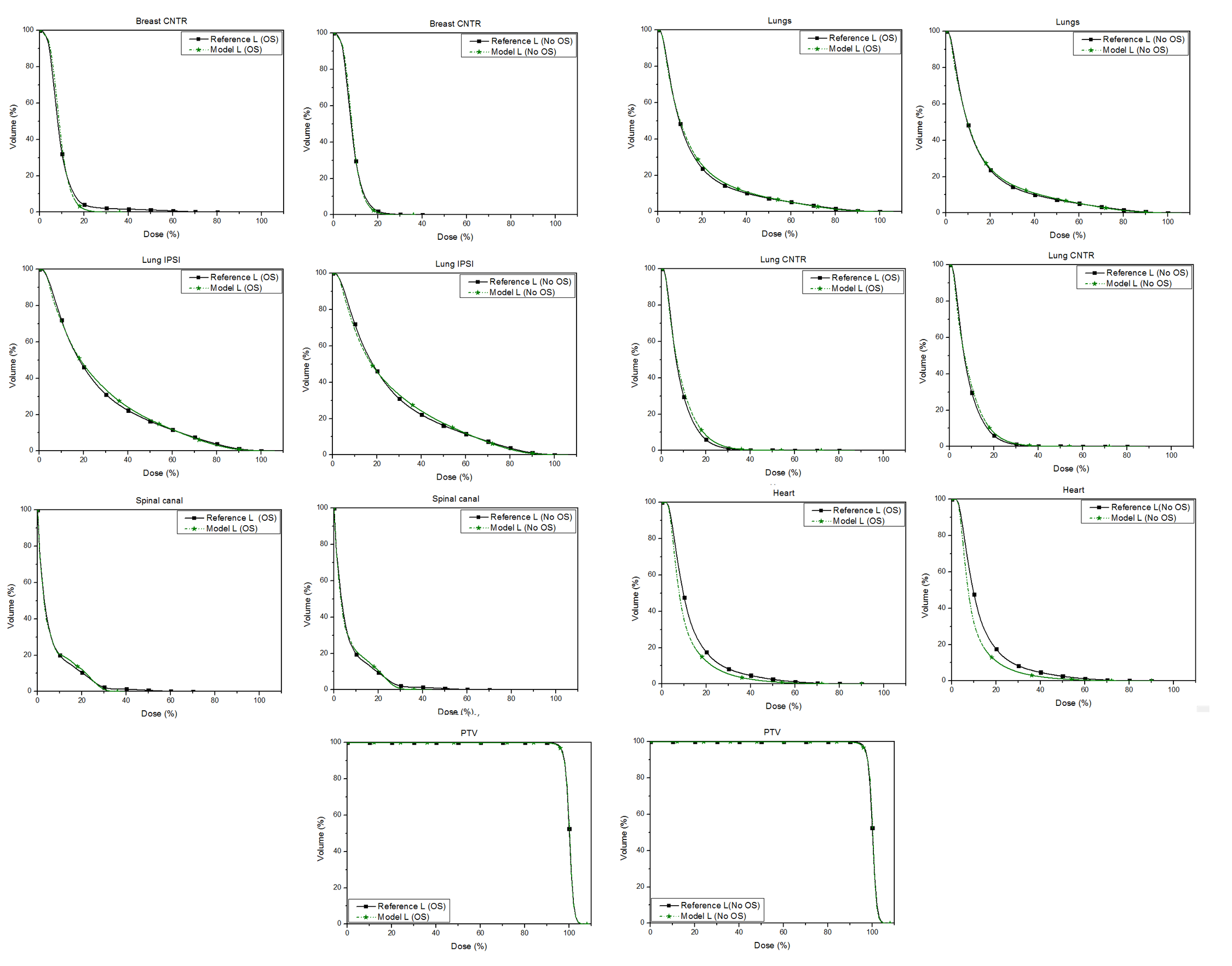

Supplement: S2 Fig — Comparison between Reference L plans in black with the square symbol and model L plans in green with the star symbol. (TIF) [file pone.0245305.s003.tif]

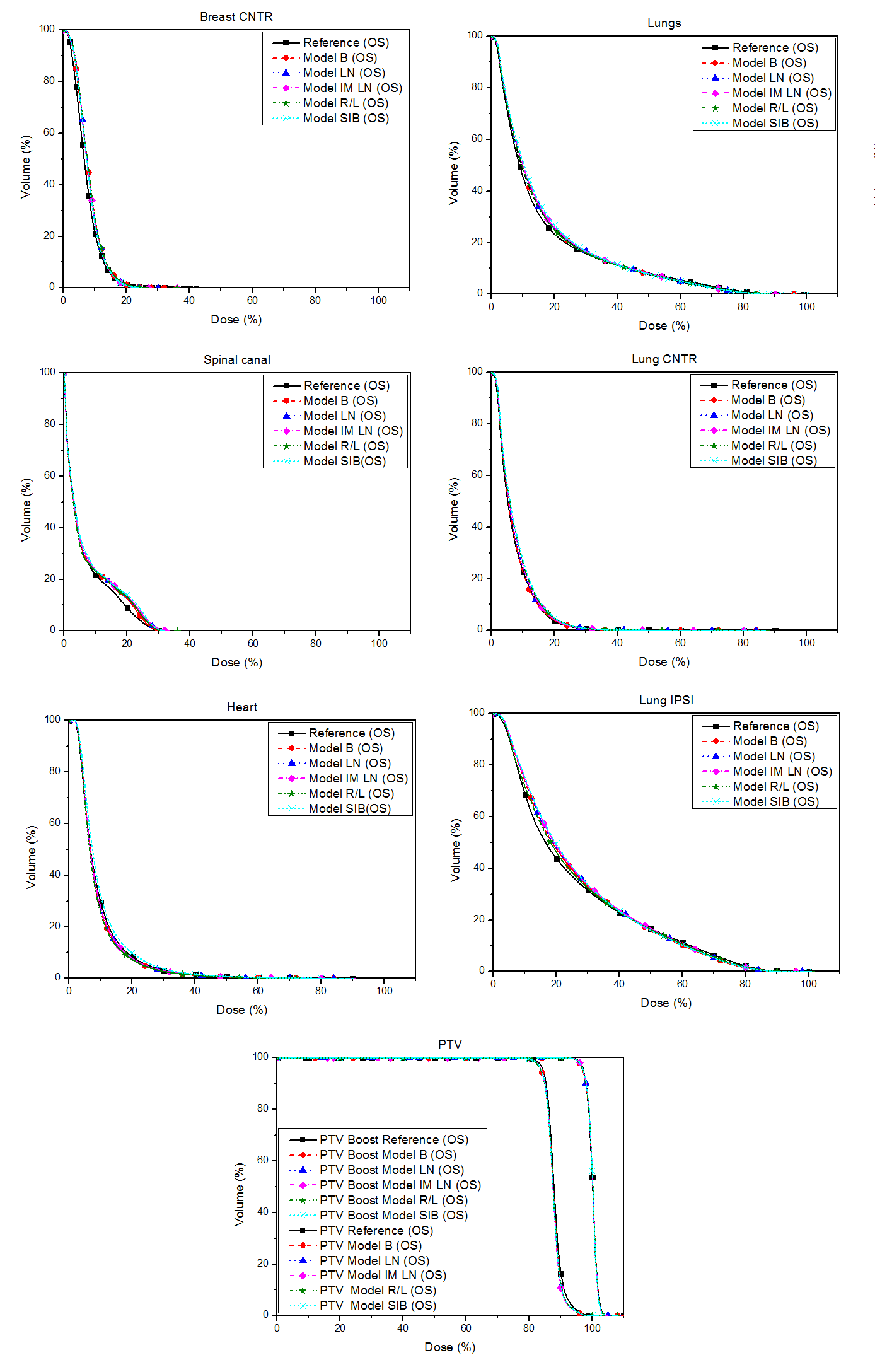

Supplement: S3 Fig — Comparison between Reference SIB plans in black with the square symbol and model B (SIB plans only) in red with the round symbol, model LN (SIB plans only) in blue with the triangle symbol, model IM LN (SIB plans only) in magenta with the rhomboidal symbol, the sum of model R and L (SIB plans only) in green with the star symbol and model SIB in cyan with the cross symbol. (TIF) [file pone.0245305.s004.tif]
